# Supplementary material for: Hierarchical organization and assembly of the archaeal cell sheath from an amyloid-like protein
Source: Nat Commun. 2023 Oct 23;14:6720. doi: 10.1038/s41467-023-42368-2 (PMC10593813; doi:10.1038/s41467-023-42368-2)
Supplement: Supplementary file 1 — Supplementary Information [file 41467_2023_42368_MOESM1_ESM.pdf]

Supplementary Figures

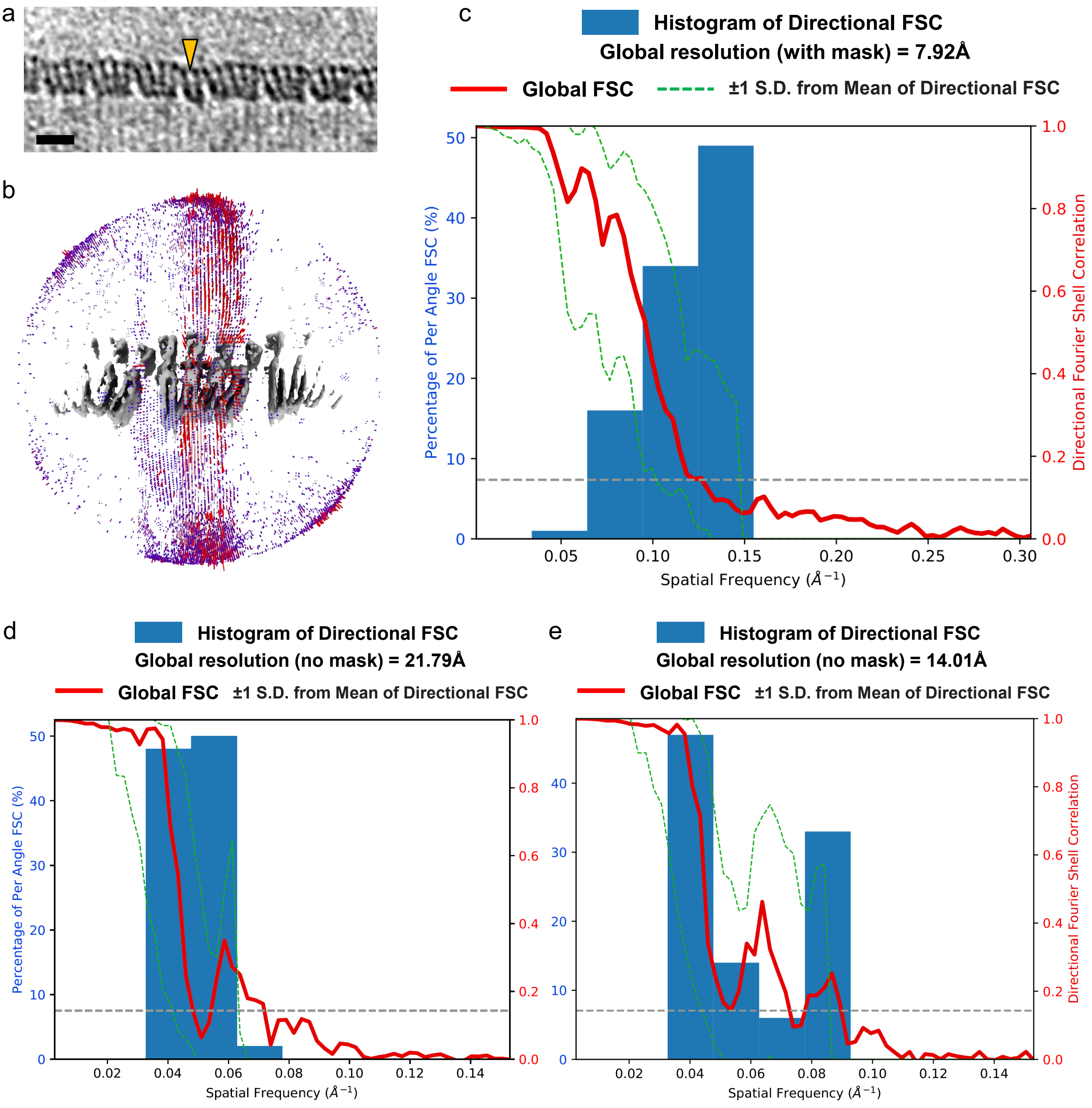

Supplementary Fig. 1: Raw cryoET tomogram and quality evaluation for subtomogram averages.

a, A density slice of cryoET tomogram with a 2-β-ring hoop indicated by the arrowhead. **b,** Particle orientation distribution. **c-e,** Directional Fourier shell correlation (FSC) curves for the subtomogram averages of 4-β-ring hoop (**c**), 3-β-ring hoop (**d**), 5-β-ring hoop (**e**). Scale bar = 10nm in **a**.

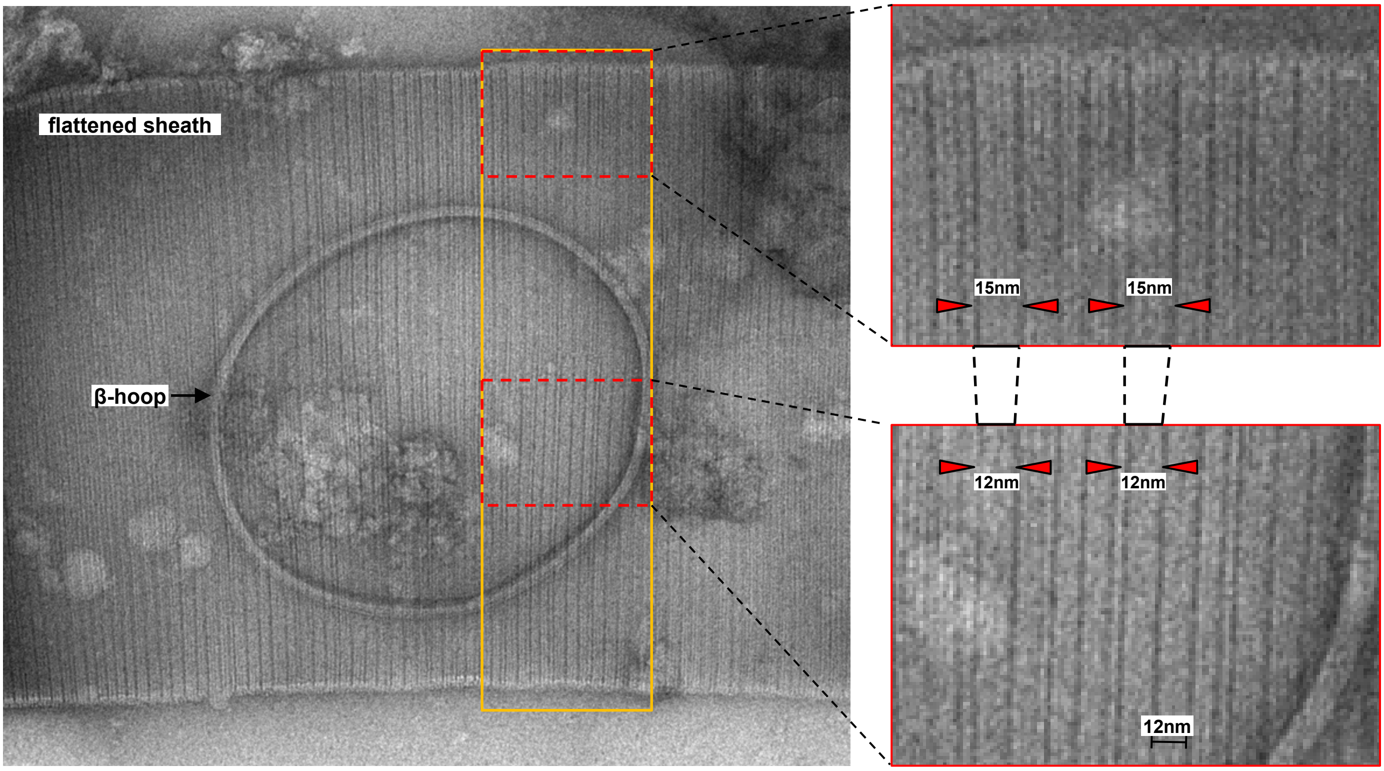

Supplementary Fig. 2: Negative stain TEM image of a region of a "ghost" *M. hungatei* cell showing overlapped hoop and flattened sheath segment. The yellow-boxed region in the left panel highlights a segment of sheath layer within which β-rings switch membership between neighboring β-hoops, as exemplified by those in the two insets. The stain-filled vertical dark strips (pointed to by red triangles) are the gaps between neighboring β-hoops. As indicated by its variable width, a β-hoop could change from a 5-β-ring-hoop (~15nm in width) to a 4-β-ring-hoop (~12nm in width).

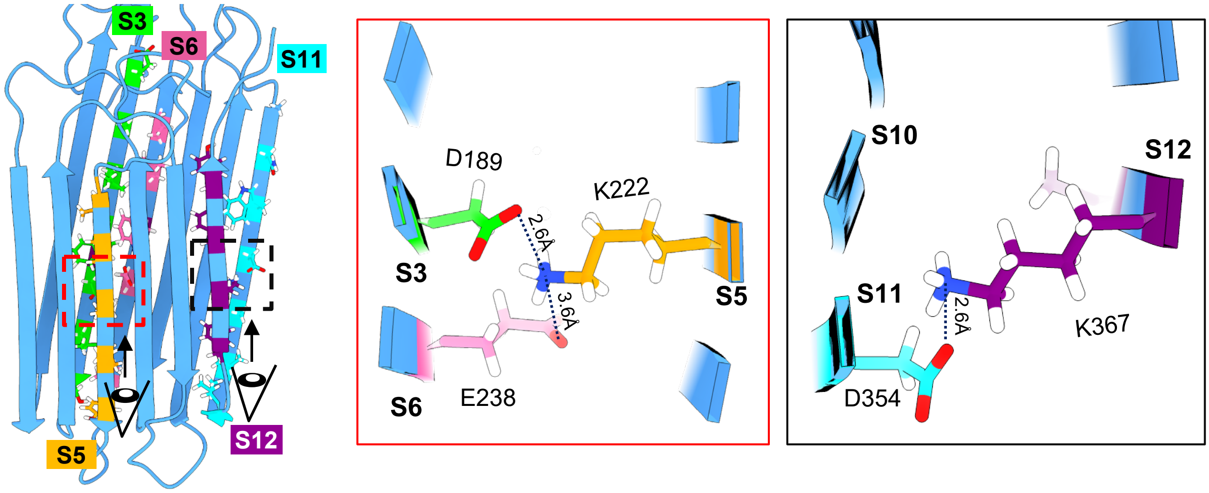

Supplementary Fig. 3: Inner connection between β-sheet 1 and 2.

Salt bridges (D189-K222 or E238-K222, and D354-K367) connect from β-sheet 1 to β-sheet 2 in the amyloid-like domain. The region and viewing orient of insets are indicated in the left guide image.

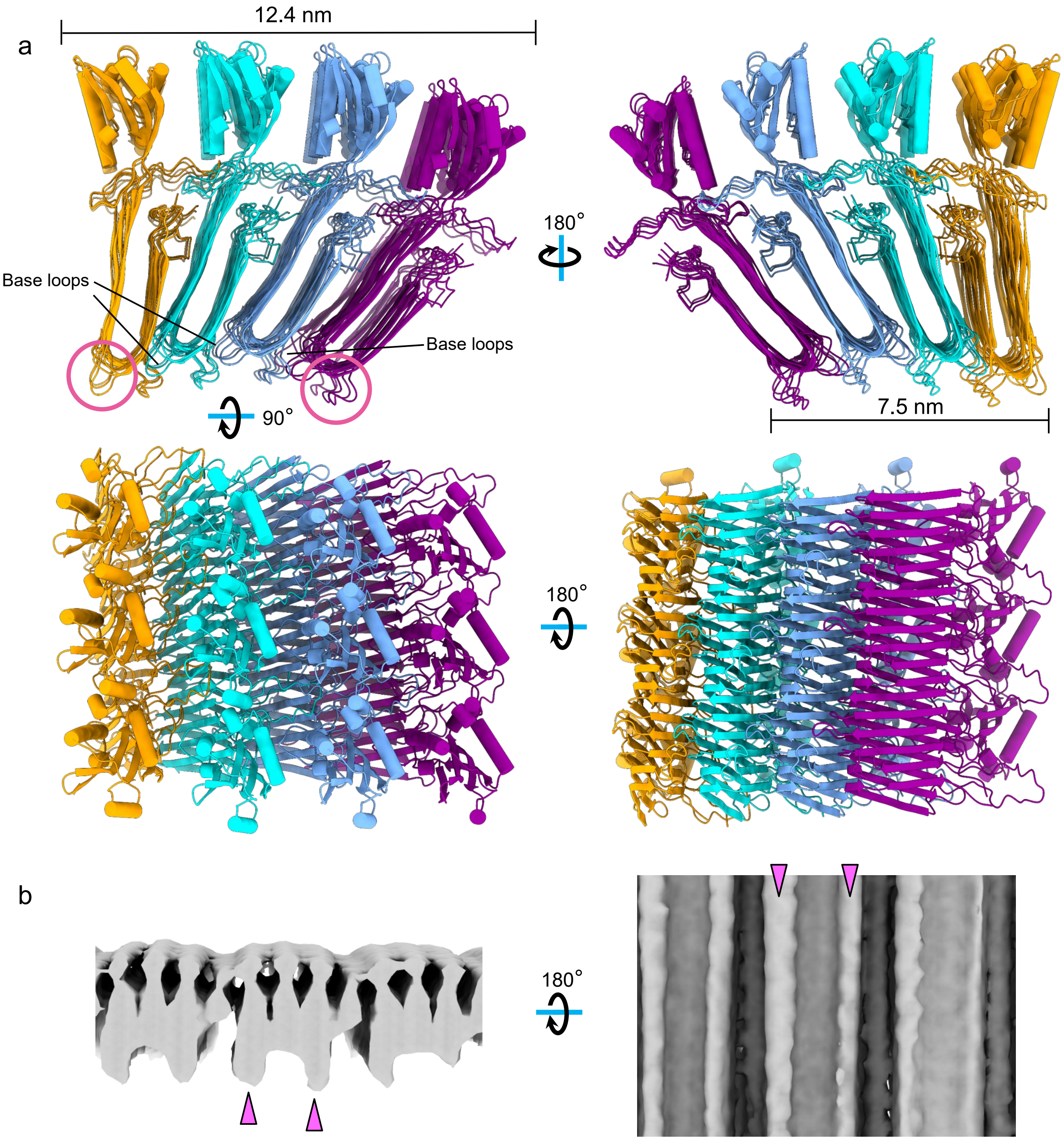

Supplementary Fig. 4: Atomic model of the 4-β-ring hoop assembly and subtomogram average of β-hoop with extra densities near the plug end.

a, Orthogonal views of a 4-β-ring hoop segment are shown as ribbon and colored by β-rings. Each β-ring segment contains 3 SH subunits. The exposed base loops on are circled both sides of the inter-hoop cleft. **b**, Orthogonal views of the subtomogram average of β-hoop near the plug end and with extra attached densities. The extra densities are indicated by pink arrowheads and their locations are correspond to the free base loops as circled in **a**.

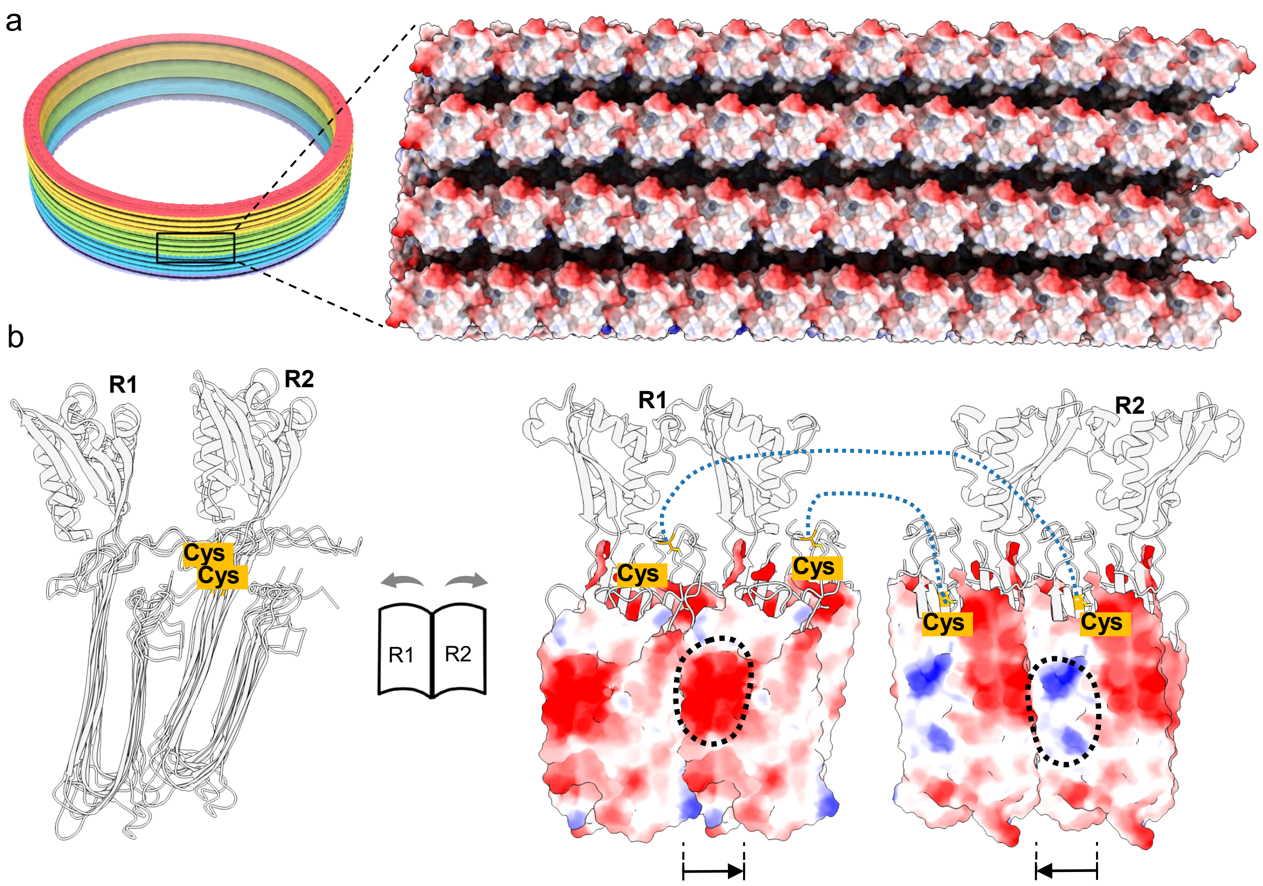

Supplementary Fig. 5: Surface charge of β-hoop.

a, A 4-β-ring hoop segment, with extensive negative (red) electrostatic potential at exterior surface. **b,** An open book view of neighboring β-rings (R1 and R2) in a β-hoop, showing the distribution of the complementary electrostatic potential at their interface (red and blue corresponds to negative and positive charges, respectively) and potential disulfide bonds as indicated by the dotted lines.

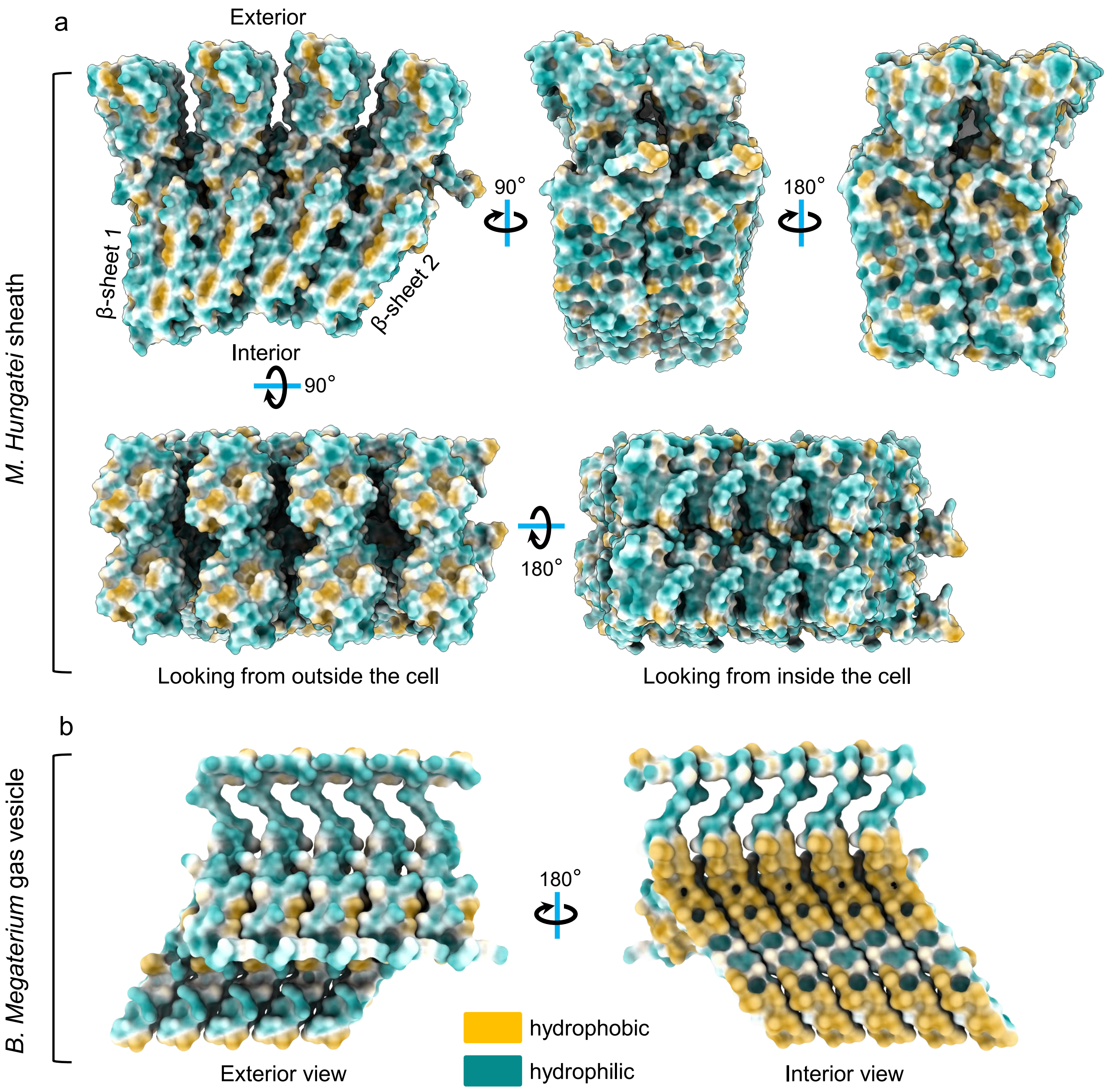

Supplementary Fig. 6: Hydrophobicity of β-hoop.

a, Orthogonal surface views of a 4-β-ring hoop segment containing 8 SH subunits, colored by hydrophobicity. **b,** a segment of the *Bacillus megaterium* gas vesicles (PDB: 7R1C), colored by hydrophobicity, shows its strong hydrophobic interior and hydrophilic exterior.
