# Supplementary material for: Hierarchical organization and assembly of the archaeal cell sheath from an amyloid-like protein
Source: Nat Commun. 2023 Oct 23;14:6720. doi: 10.1038/s41467-023-42368-2 (PMC10593813; doi:10.1038/s41467-023-42368-2)
Supplement: Supplementary file 3 — Description of Additional Supplementary Files [file 41467_2023_42368_MOESM3_ESM.pdf]

<u>**Description of Additional Supplementary Files**</u>

Legends for Supplementary Movies:

Supplementary Movie 1. Subtomogram average of segments of a 4-β-ring hoop, rendered as shaded surfaces.

Supplementary Movie 2. Assembly of the M. hungatei sheath. The cryoET density map (gray) of a cell is sliced through while montaged with a partial sheath model colored as in Figure 1e. The colored sheath model is assembled from subtomogram average of 4-β-ring hoops. The ribbon atomic model as in Supplementary Figure 4 illustrates the assembly of SH monomers into a segment of a 4-β-ring hoop, followed by elongation of such a segment in a β-ring as in Figure 1i, highlighting the generation of curvature needed for a self-limiting ring, rather than a limitless amyloid cross-β fibril.

Supplementary Movie 3. Architecture of a β-hoop segment. A partial 4-β-ring hoop is shown in different styles (ribbons, shaded surface as in Fig. 3f, electrostatic potential distribution as in Supplementary Fig. 5a, and surface hydrophobicity as in Supplementary Fig. 6a).

Supplementary Movie 4. Tomogram of an immature cell. The cryoET density map of an immature M. hungatei cell is shown as consecutive density slices. Related to Figure 4a.

Supplementary Movie 5. The 3D rendering of the immature cell. Surface representation of the tomogram of an immature M. hungatei cell colored as in Figure 4b-d highlight filamentous SH densities (blue) in the middle, between plug (gray) and S-layer (pink), and between S-layer and membrane (orange).

Supplementary Movie 6. Similar to that of Supplementary Movie 4, a second example of tomogram showing filamentous densities at the interstitial space between plugs of two neighboring cells. Related to Figure 4a.
