# Supplementary material for: Hierarchical organization and assembly of the archaeal cell sheath from an amyloid-like protein
Source: Nat Commun. 2023 Oct 23;14:6720. doi: 10.1038/s41467-023-42368-2 (PMC10593813; doi:10.1038/s41467-023-42368-2)
Supplement: Supplementary file 10 — Reporting Summary [file 41467_2023_42368_MOESM10_ESM.pdf]

nature portfolio

Corresponding author(s): Z. Hong Zhou

Last updated by author(s): Oct 6, 2023

Reporting Summary

Nature Portfolio wishes to improve the reproducibility of the work that we publish. This form provides structure for consistency and transparency in reporting. For further information on Nature Portfolio policies, see our Editorial Policies and the Editorial Policy Checklist.

Statistics

For all statistical analyses, confirm that the following items are present in the figure legend, table legend, main text, or Methods section.

n/a	Confirmed	
☐	☒	The exact sample size (*n*) for each experimental group/condition, given as a discrete number and unit of measurement
☐	☒	A statement on whether measurements were taken from distinct samples or whether the same sample was measured repeatedly
☒	☐	The statistical test(s) used AND whether they are one- or two-sided<br>*Only common tests should be described solely by name; describe more complex techniques in the Methods section.*
☒	☐	A description of all covariates tested
☒	☐	A description of any assumptions or corrections, such as tests of normality and adjustment for multiple comparisons
☐	☒	A full description of the statistical parameters including central tendency (e.g. means) or other basic estimates (e.g. regression coefficient) AND variation (e.g. standard deviation) or associated estimates of uncertainty (e.g. confidence intervals)
☒	☐	For null hypothesis testing, the test statistic (e.g. *F*, *t*, *r*) with confidence intervals, effect sizes, degrees of freedom and *P* value noted<br>*Give P values as exact values whenever suitable.*
☒	☐	For Bayesian analysis, information on the choice of priors and Markov chain Monte Carlo settings
☒	☐	For hierarchical and complex designs, identification of the appropriate level for tests and full reporting of outcomes
☒	☐	Estimates of effect sizes (e.g. Cohen's *d*, Pearson's *r*), indicating how they were calculated

Our web collection on statistics for biologists contains articles on many of the points above.

Software and code

Policy information about availability of computer code

Data collection	CryoET data were collected either with FEI Batch Tomography (lower Mag. datasets) or SerialEM 3.7 (higher Mag. dataset).
Data analysis	CryoET was processed with MotionCor2 1.26, CTFFIND 4.1, Imod 4.9.9, IsoNet v0.2, PEET 1.15 and Relion 4.0 following the workflow described in Methods. Protein atomic model prediction was performed using Alphafold2 ColabFold [https://colab.research.google.com/github/sokrypton/ColabFold/blob/main/AlphaFold2.ipynb]. CryoET data visualization and measurements were performed in Imod 4.9.9 and ChimeraX 1.4-1.6. Resolution determination was performed using 3DFSC online server.

For manuscripts utilizing custom algorithms or software that are central to the research but not yet described in published literature, software must be made available to editors and reviewers. We strongly encourage code deposition in a community repository (e.g. GitHub). See the Nature Portfolio guidelines for submitting code & software for further information.

Data

Policy information about availability of data

All manuscripts must include a data availability statement. This statement should provide the following information, where applicable:

- Accession codes, unique identifiers, or web links for publicly available datasets
- A description of any restrictions on data availability
- For clinical datasets or third party data, please ensure that the statement adheres to our policy

The subtomogram average structure data generated during the current study have been deposited in the Electron Microscopy Data Bank (EMDB) repository, with

the accession codes EMD-29442 [https://www.ebi.ac.uk/emdb/EMD-29442] (4-β-ring hoop), EMD-29443 [https://www.ebi.ac.uk/emdb/EMD-29443] (3-β-ring hoop), and EMD-29448 [https://www.ebi.ac.uk/emdb/EMD-29448] (5-β-ring hoop). The previously published structure of α-synuclein shown in Figure 2d is available in the Protein Data Bank (PDB) repository under accession code 7LC9 [https://www.rcsb.org/structure/7LC9]. The major curlin subunit predicted atomic model shown in Figure 2d is available in AlphaFoldDB with accession code AF-P28307-F1 [https://alphafold.ebi.ac.uk/entry/P28307]. The atomic model of Bacillus megaterium gas vesicle segment shown in Supplementary Figure 6 is available in PDB with accession code 7R1C [https://www.rcsb.org/structure/7R1C].

Human research participants

Policy information about studies involving human research participants and Sex and Gender in Research.

Reporting on sex and gender	N/A
Population characteristics	N/A
Recruitment	N/A
Ethics oversight	N/A

Note that full information on the approval of the study protocol must also be provided in the manuscript.

Field-specific reporting

Please select the one below that is the best fit for your research. If you are not sure, read the appropriate sections before making your selection.

☒ Life sciences ☐ Behavioural & social sciences ☐ Ecological, evolutionary & environmental sciences

For a reference copy of the document with all sections, see nature.com/documents/nr-reporting-summary-flat.pdf

Life sciences study design

All studies must disclose on these points even when the disclosure is negative.

Sample size	Three cryoET datasets were collected in this work. There are 10, 23 and 37 tomograms collected and reconstructed for the higher magnification (Whole cell), lower Magnification (Whole cell), lower Magnification (Immature cell), respectively.<br>Subtomogram average: 2971, 17359 and 3609 particles were extracted from 10 tomograms in higher Magnification (Whole cell) dataset and used for the final 3D reconstruction for the structure of 3, 4, 5-β-ring hoops, which are sufficient to get the reported resolutions.
Data exclusions	CryoET particles which have poor qualities or are not interested targets were excluded during 3D classification. This standard procedure has been widely used to obtain higher resolution structures of macromolecules.
Replication	M. hungatei strain JF1 was cultured following the published protocol for the above mentioned three datasets, independently. Before each of the data collection session, one or more negative stain imaging check were performed to make sure the experiment is reproducible and the sample looks good. The replicates were not feasible for cryoET.
Randomization	Randomization was not relevant for the experiments performed here. Before the data collection, negative stain imaging was performed to make sure the cells were reproducible and their density on the EM grids was good. During the cryoET data collection, we only selected areas containing cells for imaging, no other restriction was applied. In the cryoET data processing, all tomograms with clear sheath density were used for subtomogram average, no other restriction was applied.
Blinding	Blinding was not relevant for this study. The major conclusions of this study are based on the direct observation of tomograms or subtomogram averages structure data which are not relevant to whether the researchers were blinded to the biological sample.

Reporting for specific materials, systems and methods

We require information from authors about some types of materials, experimental systems and methods used in many studies. Here, indicate whether each material, system or method listed is relevant to your study. If you are not sure if a list item applies to your research, read the appropriate section before selecting a response.

Materials & experimental systems

n/a	Involved in the study
☒	☐ Antibodies
☒	☐ Eukaryotic cell lines
☒	☐ Palaeontology and archaeology
☐	☒ Animals and other organisms
☒	☐ Clinical data
☒	☐ Dual use research of concern

Methods

n/a	Involved in the study
☒	☐ ChIP-seq
☒	☐ Flow cytometry
☒	☐ MRI-based neuroimaging

Animals and other research organisms

Policy information about studies involving animals; ARRIVE guidelines recommended for reporting animal research, and Sex and Gender in Research

Laboratory animals	This study did not involve laboratory animals.
Wild animals	No wild animals are used in this study.
Reporting on sex	Archaeal cells are asexual and this sex consideration does not apply in this study.
Field-collected samples	This study did not involve samples collected from the field.
Ethics oversight	All of the related experiments were performed in the Department of Microbiology, Immunology, and Molecular Genetics at UCLA, and the UCLA-DOE Institute. No ethics guidance is required for this study.

Note that full information on the approval of the study protocol must also be provided in the manuscript.
